# A Penetrating Neck Injury Resulting in the Complete Transection of the Ipsilateral Common Carotid Artery, Delayed Contralateral Pneumothorax, and Occult Esophageal Injury: A Case Report With a Multidisciplinary Approach to Management

**DOI:** 10.7759/cureus.56988

**Published:** 2024-03-26

**Authors:** Christian Przeslawski, Michaela Knaggs, Peter M Habib, Ahmed Ghamraoui, Caitlin Wahl, Jeffrey Gerken

**Affiliations:** 1 General Surgery, Beaumont Hospital - Farmington Hills Campus, Farmington Hills, USA; 2 Osteopathic Medicine, Michigan State University College of Osteopathic Medicine, East Lansing, USA; 3 Vascular Surgery, Beaumont Hospital - Farmington Hills Campus, Farmington Hills, USA; 4 Thoracic Surgery, Beaumont Hospital - Farmington Hills Campus, Farmington Hills, USA

**Keywords:** penetrating esophageal injury, penetrating trauma with contralateral pneumothorax, internal jugular transection, internal jugular ligation, common carotid transection, common carotid ligation, penetrating neck trauma

## Abstract

A 29-year-old male presented with a zone one penetrating neck injury resulting in complete transection of the left carotid sheath and its contents. The proximal common carotid artery and internal jugular vein injuries were successfully managed with vessel ligation without adverse neurological sequelae. The patient also developed a contralateral pneumothorax, which was due to an occult through-and-through esophageal injury at the junction of the cervical and thoracic esophagus. The esophageal injury was successfully managed with surgical repair and wide drainage of the neck and right chest.

## Introduction

Penetrating trauma frequently has unpredictable trajectories, resulting in injuries distant from the original point of entry. This concept holds especially true with high-velocity ballistic injuries; however, it is not always as evident with low-velocity injuries such as knife wounds [1]. Regardless of the mechanism, continuous thorough reevaluation of trauma patients is paramount in identifying occult injuries not apparent on initial surveys and imaging.

Additionally, penetrating trauma to the proximal neck presents a unique challenge. This transition point between the neck and thoracic cavity often necessitates highly morbid exploration in order to adequately expose the aortic arch vessels, central veins, and vital aerodigestive structures that traverse this region, such as the carotid artery and esophagus. Whenever possible, injuries to the internal and common carotid artery should be repaired or shunted at the index operation to avoid devastating cerebrovascular ischemic injury. Esophageal injuries are also historically taught to be repaired primarily via laparotomy, thoracotomy, or neck exploration, depending on the location [2]. There is little data on how to manage injuries at the junction points, as such cases are morbid and extremely technically challenging. Herein, we present an interesting case of a penetrating neck injury resulting in the ipsilateral common carotid artery and internal jugular vein transection, as well as an occult esophageal perforation with contralateral pneumothorax. This case discusses multiple interesting trauma concepts helpful for trauma surgeons in training to understand.

## Case presentation

A 29-year-old male was dropped off by a private vehicle after an assault with a stab wound to the left neck. The patient had pulsatile bleeding from the neck wound, which was approximately 2 cm in length. The patient was noted to be in extremis. Direct pressure was held over the left neck as the patient was intubated. During intubation, a small amount of blood in the oropharynx was noted, without other obvious injury to the oropharynx being visualized. Advanced Trauma Life Support (ATLS) protocol was followed, and massive transfusion was initiated. The patient had clear and equal bilateral breath sounds. He did have five other superficial stab wounds to the bilateral upper extremities extending into the subcutaneous tissue without active bleeding. Focussed Assessment with Sonography in Trauma (FAST) exam was negative for free fluid. Chest radiograph was unremarkable.

Due to the severity of the injury and the inability to control hemorrhage in the trauma bay, the patient was emergently brought to the operating room with continued pressure held over the left neck. After prepping and draping, the wound was observed to be located directly posterior to the sternocleidomastoid muscle underneath the level of the cricoid cartilage, consistent with a zone one penetrating neck injury. The wound was extended medially, similar to a collar incision. Further dissection revealed a complete transection of the left internal jugular vein and common carotid artery just proximal to the bifurcation of the internal and external carotid artery with a massive amount of hemorrhage. There was a significant retraction of the common carotid artery into the chest. Clamps were able to be placed on the proximal and distal common carotid stumps. The proximal and distal segments of the internal jugular vein were identified and suture ligated with 0-silk suture. Brisk back-bleeding was demonstrated from the distal common carotid stump which was approximately two centimeters from the bifurcation to the internal and external carotid arteries. The estimated blood loss from the neck wound was 2.5 liters; however, the hemorrhage had been controlled at this point. The patient remained significantly hemodynamically unstable, requiring continued massive transfusion. Due to the ongoing resuscitation requirements and the proximal location of the carotid injury, the decision was made to proceed with ligation of the common carotid artery, and a median sternotomy was not performed. This decision was made knowing that the patient had demonstrated significant collateral flow with brisk back-bleeding from the distal carotid artery stump. Placing a shunt was discussed, but for similar reasons stated above-including adequate collateral circulation and technical difficulty placing the shunt-it was decided to forego such intervention. The proximal and distal ends of the left common carotid artery were then suture-ligated in a two-layer fashion with a 5-0 prolene suture. The first layer was thrown in horizontal mattress fashion, followed by a second oversewing layer, which ran as a continuous suture. The wound was packed with iodine and loosely closed with staples. Before leaving the operating room, a nasogastric tube was inserted with approximately 1 liter of bloody gastric fluid immediately suctioned from the stomach. Esophagogastroscopy was immediately performed, and demonstrated remnant food and dark blood; however, no obvious mucosal defect or active bleeding was observed. There was no blood upon suctioning the endotracheal tube and therefore bronchoscopy was not performed.

**Figure 1:**
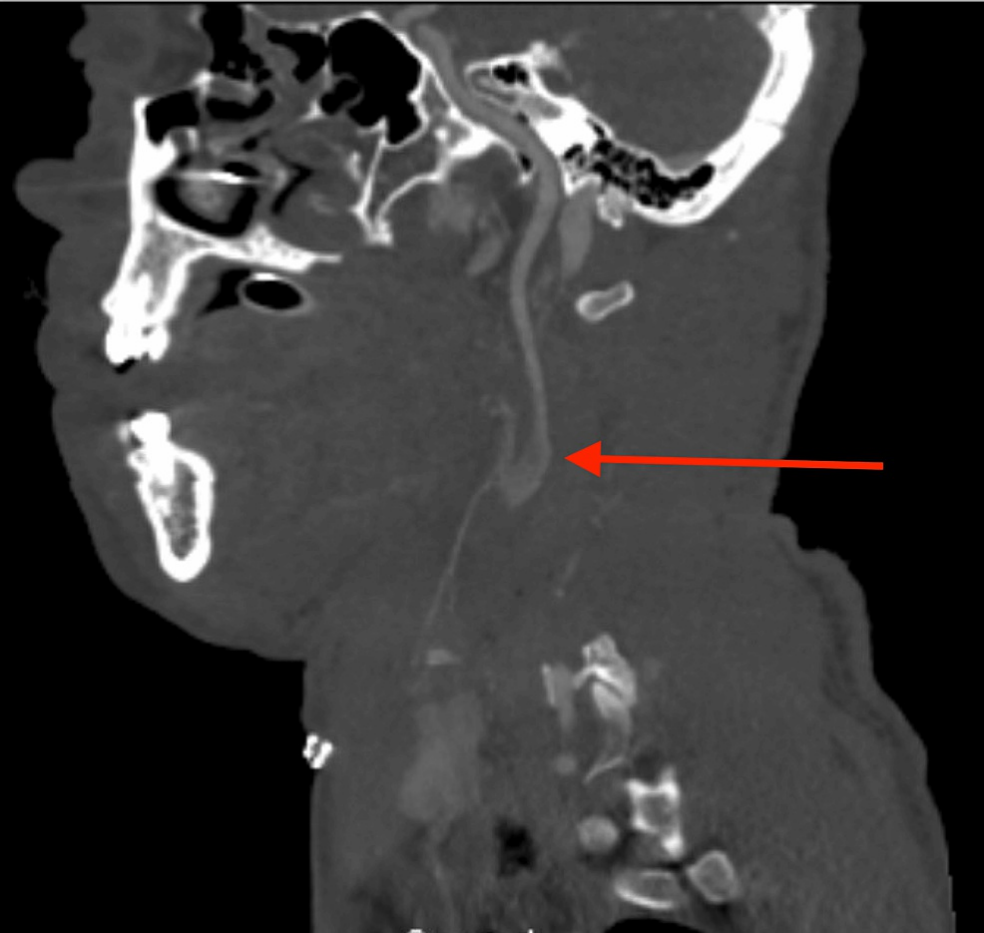
CT angiography of the neck in sagittal views, demonstrating backflow through the external carotid artery up into the internal carotid artery

**Figure 2:**
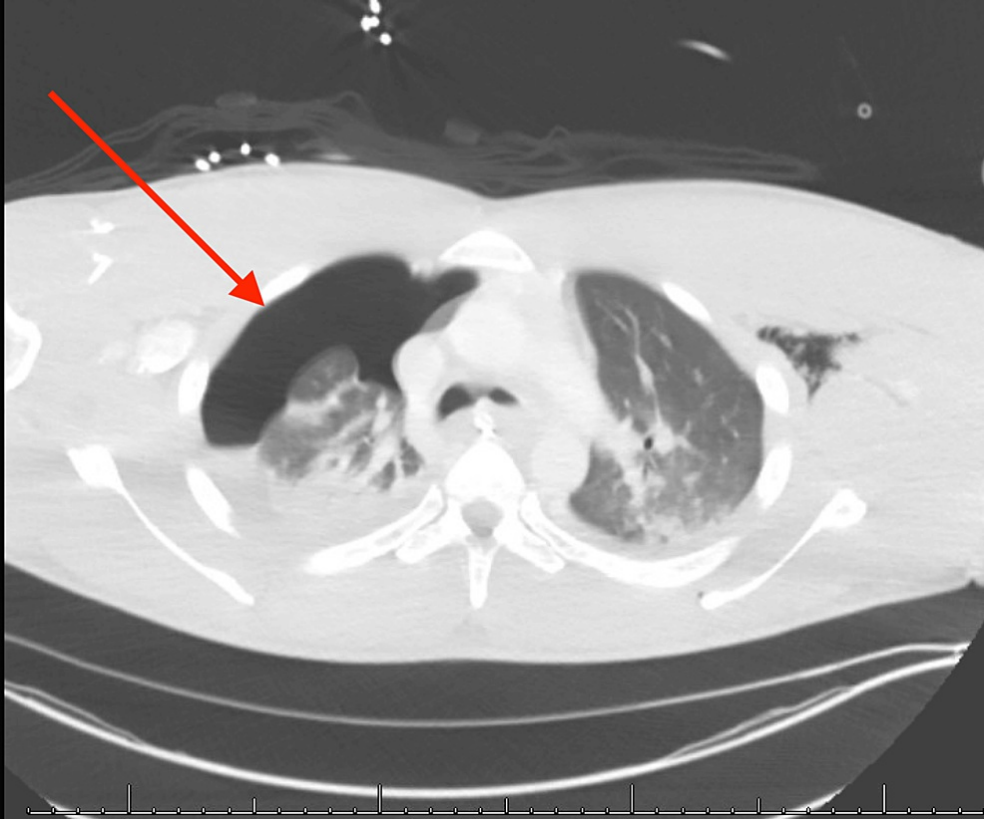
Large right pneumothorax on CT axial view of the thorax

The patient was then transferred to the surgical intensive care unit (SICU) with ongoing resuscitation. In total, the patient received a total of 12 units of packed red blood cells, 12 units of fresh frozen plasma, and two packs of platelets. After receiving these blood products, the patient was stable and was able to undergo computed tomography (CT) imaging, including CT angiography of the head and neck, which revealed adequate collateral circulation to the left side of the brain (Figure 1). Surprisingly, the right chest was found to have a massive pneumothorax (Figure 2). A right-sided thoracostomy tube was placed with immediate decompression of the pneumothorax. There was no change in hemodynamics or ventilatory status associated with this intervention.

The following day, the thoracic surgery team took the patient for repeat esophagogastroduodenoscopy (EGD) and neck re-exploration due to concern for missed esophageal injury in the setting of contralateral pneumothorax. Upon return to the operating room, gastroscopy found old blood in the stomach, as well as blood trickling from a left cervical esophageal full-thickness wound. An area of mucosal irregularity of the right intraluminal side of the esophagus was observed during EGD. While re-exploring the left neck wound, an esophageal injury was identified on the esophagus in the left chest at approximately the vertebral level of T1. This defect was closed in a standard two-layer fashion with a strap muscle buttress for reinforcement. The skin incision was extended to the right side of the neck in a collar fashion, and the right side of the esophagus was thoroughly examined. Despite dissection of the esophagus down to the level of the sternal notch, no injury was appreciated. When the gastroscope was reintroduced, it was noted that the right-sided mucosal defect was below the thoracic inlet at approximately T2 level. Blunt dissection was able to be continued down into the mediastinum to this point, but again, there was no perceivable full-thickness injury. A leak test was performed, and no obvious leak was appreciated in the neck, nor was there an air leak visible on the right-sided chest tube. As there was no confirmation of a full-thickness injury on the right, wide drainage was elected rather than proceeding with an exploratory thoracotomy in order to spare the patient from the morbidity of a median sternotomy. A drain was placed through the neck to this area, and the right intrapleural chest tube was already in position to drain the right thoracic cavity. Bronchoscopy was not performed as the trajectory towards the trachea was not evident. Blake drains were placed in the bilateral tracheoesophageal grooves, and the patient was returned to the SICU. The patient was then started on vancomycin, cefepime, flagyl, and micafungin prophylactically. He was also initiated on aspirin 81 mg.

On hospital day three, the patient self-extubated and was noted to be in severe respiratory distress. He was urgently reintubated. In consultation with the otolaryngology team, controlled extubation over a Cook catheter with fiberoptic laryngoscopy was attempted on hospital day six, which revealed bilateral vocal cord paralysis. Ultimately, on hospital day 11, the patient had an open tracheostomy and percutaneous endoscopic gastrojejunostomy (GJ) tube placed as he had likely experienced bilateral recurrent laryngeal nerve injuries as a result of either the initial injury or during operative intervention. The gastric lumen of the GJ-tube was kept to gravity, with the jejunal lumen used for feeds, in an attempt to decrease reflux that could damage the wounds and recent esophageal repair. Ampicillin-sulbactam was started, and the remainder of the patient's antibiotics were discontinued.

**Figure 3:**
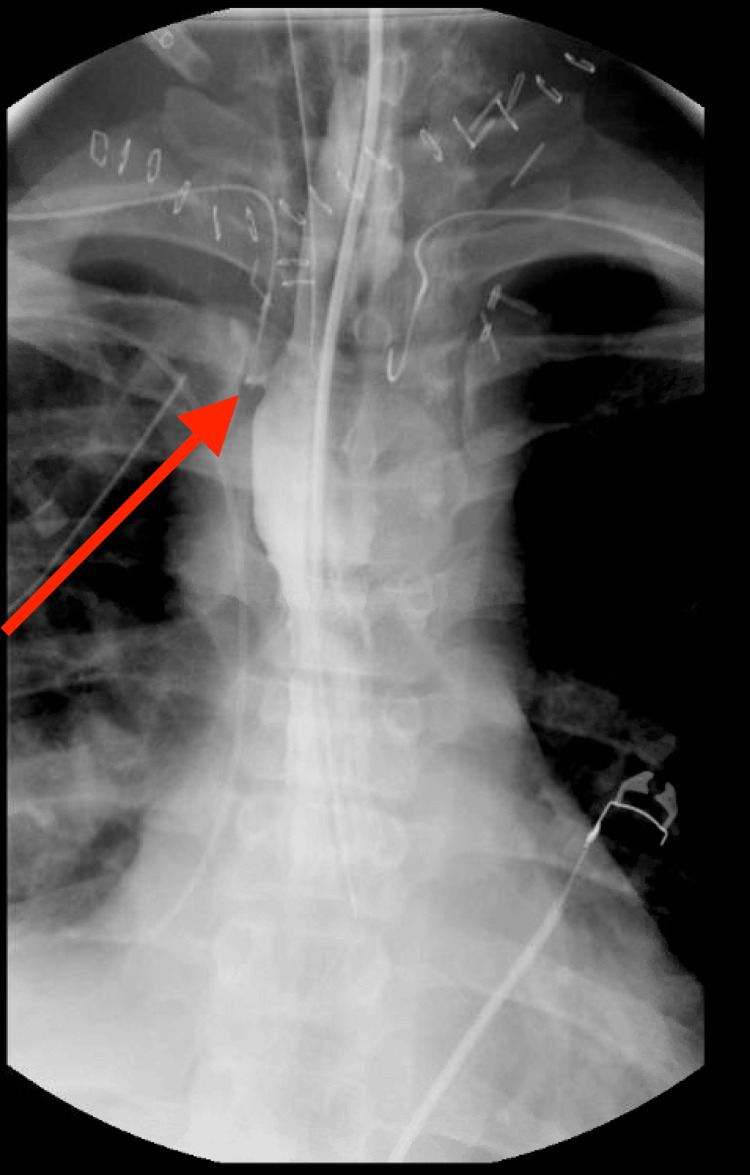
Small right-sided esophageal leak on hospital day seven near the cervicothoracic junction

**Figure 4:**
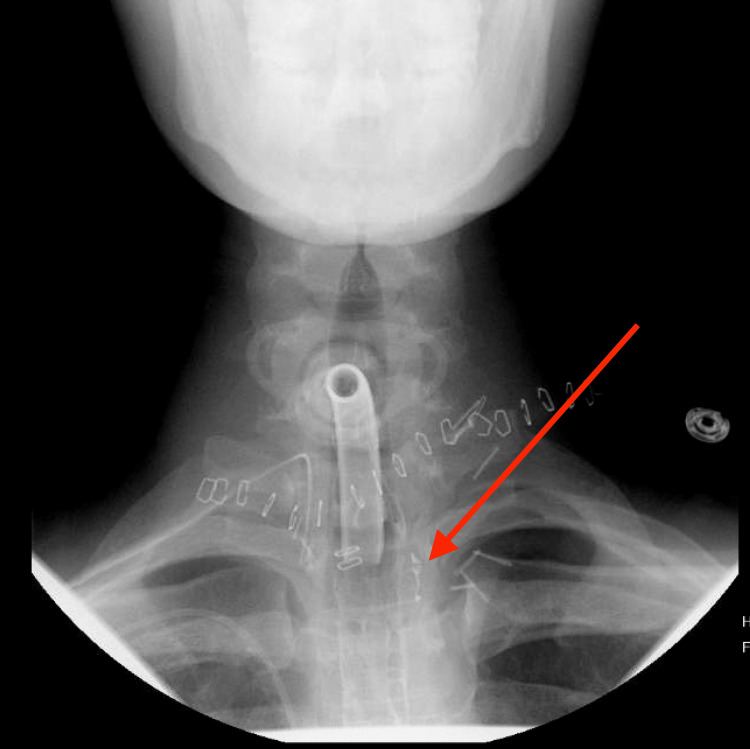
Left-sided contained esophageal leak on day 33

Multiple barium esophagrams were performed during the patient's admission. On hospital day seven, the esophagram demonstrated a small right-sided contrast leak at approximately the level of T2 (Figure 3). Further operative or endoscopic intervention was not performed as the leak was already well-controlled by a previously placed surgical drain, and the patient was afebrile without leukocytosis. The chest tube was removed on hospital day 13. Ampicillin-sulbactam was discontinued on hospital day 15. On day 20, a repeat esophagram demonstrated the resolution of the right-sided leak, and the right cervical drain was removed. However, new contrast extravasation from the left esophagus near the clavicle was visualized at the level of C7, suggestive of the breakdown of the prior repair (Figure 4). The left-sided leak appeared contained to the cervical region and was managed with observation. The patient did not develop any signs or symptoms suggestive of infection. An esophagram on day 33 showed the resolution of the left esophageal leak. The patient was discharged from the hospital after a 36-day admission.

The patient was last seen in the thoracic surgical office 10 weeks after the initial presentation. He was noted to be improving well, although he remained tracheostomy dependent. He had been tolerating a regular diet, and his gastrojejunostomy tube was removed in the office. He followed up in the vascular surgery office 11 weeks after the initial surgery, at which time there were no neurologic deficits. It was recommended he continue daily aspirin indefinitely.

## Discussion

Penetrating injury to the great vessels and their primary branches is a common cause of early mortality in trauma patients. Internal jugular ligation in the setting of penetrating neck trauma is typically well-tolerated when the contralateral internal jugular vein is intact. However, the Eastern Association for the Surgery of Trauma (EAST) guidelines recommend carotid injuries undergo reconstruction unless the defect is a small intimal injury alone [2]. Hemodynamic stability must be a factor in decision-making during surgical repair of such injuries, and occasionally, ligation is the only means of stabilizing a patient. In cases where the patient is stable enough to consider different management options, there is not an established protocol on which procedure produces the best results. Kuehne et al. found that the majority of patients with internal carotid artery injuries did not have an improvement in neurological status despite whether their injuries were managed with repair, ligation, or observation [3]. Another important therapeutic measure to consider is the placement of the intravascular shunt. Intravascular shunts are temporary measures to restore blood flow before attaining definitive management. Asencio et al. performed a 2021 review of published studies on patients with common and internal carotid artery trauma, examining outcomes of those who underwent shunting versus those who did not. This review found that shunted patients had improved outcomes in maintaining or increasing neurologic status; however, there was no statistically significant difference in mortality between the two groups. They recommended that shunting should be considered in carotid artery injury patients [4]. This review did not include patients managed with ligation. Performing a shunt was considered for the patient presented in this case during his index surgery, though we decided to forego shunting for the same reason repair was not performed. The backbleeding was sufficient, and a shunt would have been extremely difficult to secure on the proximal carotid artery, likely requiring median sternotomy. The patient, at this point, was still being actively resuscitated at that time.

There is an important distinction between tolerance of common carotid versus internal carotid ligation. In a retrospective review by Ramadan et al. on common and internal carotid injuries over a six-year period, they identified that common carotid ligation resulted in a mortality rate of 11% while internal carotid artery mortality rate was 21%. This review also showed that all four of the deaths occurring after common carotid artery injury were secondary to exsanguination, while 80% of the deaths following internal carotid artery injury occurred secondary to neurologic decline [5]. Stroke risk is of great concern in any carotid trauma, especially when ligation is performed. Ramadan et al. note that in cases of internal or common carotid artery trauma, operative ligation results in stroke 57% of the time. Stroke rates for common carotid and internal carotid ligation are 11% and 41%, respectively. Stroke rates are 56% and 16% for patients presenting with blunt and penetrating carotid trauma, respectively, which is a significant difference. However, mortality is more common with penetrating trauma than blunt, with 12 of 14 deaths in this review resultant of penetrating injury. A principle prognostic indicator is a patient's neurologic status upon presentation to the hospital. All patients with a GCS above eight did not suffer mortality. Patients who did present with a GCS of eight or below (comatose) had a 50% mortality rate. For patients who presented neurologically intact and subsequently underwent carotid ligation, there was 0% mortality and 50% without permanent deficit [5]. In this patient's case, he was neurologically intact on arrival with a penetrating neck injury, prognosticating a low risk of stroke. The lesion was extremely proximal on the common carotid, and ligation of the left common carotid was performed due to his ongoing hemodynamic instability. Excellent back bleeding was observed intraoperatively from the distal common carotid artery stump. Following surgery, a CT angiogram of the head and neck was performed, which showed adequate circulation throughout the circle of Willis, as well as flow through the left superior thyroid, external carotid, and internal carotid arteries. Our patient remained without stroke or neurologic deficit during admission and at follow-up. In select cases where the patient is in continued extremis and there is evidence of good collateral flow, common carotid ligation may be warranted, as control of hemorrhage is paramount in preventing mortality. All efforts should be made to immediately control and reverse hemorrhagic shock, as being in a shock state is a prognostic factor with poor outcomes in and of itself for patients undergoing ligation. Moore et al. demonstrated that for patients who required carotid artery ligation, those who were in shock at the time of surgery had a stroke rate of 66% and a mortality rate of 57% [6]. The patient presented in this case report was undoubtedly in shock throughout the surgery and received a massive transfusion as a temporizing measure while source control of the bleeding was obtained.

The delayed contralateral pneumothorax highlights several interesting points. The trajectory of high-energy injuries is notorious for its unpredictability. However, even a blade can have unexpected injuries, as discussed by Gupta et al. [1]. In regards to this case, the entry point was the left neck, with the knife traversing through and through the esophagus before entering into the right chest. Similar types of injuries have been described in the literature. Olutu et al. presented a case of a penetrating left neck injury leading to a right pneumothorax. Bronchoscopy and EGD were negative for airway or esophageal injury, though that patient did develop a chylothorax, which resolved by day 10 [7]. Another related case from Sharma et al. discussed a right supraclavicular stabbing, with CT showing air extending from the entry site retrosternal to the left hemithorax. Again, the patient had thoracic injuries contralateral to the original entry site, with left pneumothorax and delayed hemothorax due to a transected left internal mammary artery [8]. Our patient also presented with delayed pneumothorax. There was no evidence of pneumothorax on the initial primary survey or radiography. A large pneumothorax was later identified on the CT chest after the index operation was performed. Likely, this pneumothorax developed during intraoperative EGD when the esophagus and stomach were insufflated for visualization resulting in air leakage through the right esophageal injury into the right hemithorax. It is also possible there was a parenchymal injury to the lung that developed into a pneumothorax with positive pressure ventilation, though this is a less likely mechanism. Delayed pneumothoraces are not well documented in the literature for penetrating trauma. In a review of thoracic trauma, Shorr et al. noted that delayed pneumothoraces are more often seen in the setting of blunt thoracic trauma [9]. Nonetheless, it is essential to remain vigilant during the evaluation and reevaluation of trauma patients, as well as recognize the elusiveness of penetrating injury trajectories.

Esophageal injuries in trauma are most commonly caused by penetrating cervical trauma, as noted by Petrone et al. [10]. EAST guidelines recommend for primary repair of esophageal injuries [2]. The Western Trauma Association also recommends cervical and thoracic penetrating wounds be repaired when able and selective non-operative management be pursued with caution. They discuss literature that exists for non-operative management of iatrogenic esophageal injuries. Some of these injuries have been managed with endoscopic clipping and stenting [11]. Mubang et al. discussed in a 2021 review of esophageal trauma that esophageal injuries can be managed nonoperatively if the leaks are contained, the patient has mild symptoms or there are no signs of sepsis from the injury, and if the repair is expected to have a high rate of morbidity or mortality [12]. In regards to the treatment of this patient's esophageal injury, the left-sided injury was managed operatively as it was a full-thickness injury easily identified on reexploration in a controlled setting and closed in the usual two-layer fashion. The right-sided esophageal defect identified on repeat EGD was noted to be a small mucosal irregularity. Due to its location near the thoracic outlet, it would be difficult and highly morbid to access from a cervical, video-assisted laparoscopic, or open thoracotomy approach. Additionally, a right chest tube had already been placed and was adequately draining the thoracic cavity. For those reasons, it was decided that a further surgical approach was not necessary. The leak on hospital day 20 was likely secondary to moderate vascular compromise and tissue breakdown secondary to the carotid injury. This leak was a contained leak in the cervical region and self-resolved. Other than on initial presentation, the patient was hemodynamically stable. In addition, he remained afebrile and exhibited no signs of systemic sepsis. Therefore, the patient was successfully managed nonoperatively for the right-sided injury and later on for his left-sided contained leak. Another therapeutic option worth discussing is esophageal stenting, as it is a growing therapeutic option for esophageal perforations. As reported by Mubang et al., stenting can potentially be used in the setting of thoracic esophageal injuries, though it is contraindicated for cervical esophageal injuries due to the high propensity for stent migration. Additionally, stenting is not recommended when there are concomitant injuries, making it less useful in a trauma situation such as in the case of this patient [12]. Another potential consideration worth mentioning is endoscopic negative pressure therapy (ENPT), as discussed by Loske and Müller. Though uncommon in traumatic situations, these devices can be placed by specialized gastroenterologists or surgeons. The procedure involves placing a wound vacuum endoscopically over a contained esophageal injury. This potentially may have aided in quicker recovery of the bilateral esophageal defects; however, this therapy would likely have been difficult due to the right-sided chest tube which would have detracted the ability to maintain adequate suction in the ENPT system [13]. ENPT does show promise for future small esophageal injuries in institutions with advanced endoscopic capabilities. In this case and in other select cases of traumatic esophageal injury, patients can be managed effectively with wide drainage, avoiding highly morbid procedures.

## Conclusions

This case was an intriguing example of a proximal carotid injury that was managed safely with ligation without any neurologic sequelae. In addition, the case highlights why continued reevaluation is essential in trauma patients, as the trajectory of the blade, in this case, led to contralateral pathologies with delayed presentation. This case also shows that drainage of an esophageal injury in the cervicothoracic junction can be managed safely in certain situations with minimal sequelae, thus avoiding the morbidity of a large invasive thoracic procedure.
